# Comparison of Silicon-Evoked Responses on Arsenic Stress between Different Dular Rice Genotypes

**DOI:** 10.3390/plants10102210

**Published:** 2021-10-18

**Authors:** Mohammad Reza Boorboori, Zhou Li, Xue Yan, Mu Dan, Zhixing Zhang, Wenxiong Lin, Changxun Fang

**Affiliations:** Fujian Provincial Key Laboratory of Agroecological Processing and Safety Monitoring, College of Life Sciences, Fujian Agriculture and Forestry University, Fuzhou 350002, China; m.boorboori@yahoo.com (M.R.B.); chqhlj@163.com (Z.L.); yanxue@fafu.edu.cn (X.Y.); mudan@fafu.edu.cn (M.D.); zhangzhixingfz@163.com (Z.Z.)

**Keywords:** *Oryza sativa* L., molecular and physiological activities, arsenic stress, silicon

## Abstract

Arsenic is one of the most hazardous metalloids in nature, and due to its high water solubility, it is one of the most important causes of pollution. However, silicon reduces the uptake and transport of arsenic in rice. This study investigates the interaction of different arsenic and silicon levels on dry weight, protein content, and concentrations of arsenic and silicon in two different rice shoots and roots of Dular wild-type (DU-WT) and Dular Lsi1-overexpressed (DU-OE) rice. It should be noted that all seedlings were subjected to four different treatments. For RNA-seq and qPCR, the DU-WT genotype was selected as the control and DU-OE as the treatment. With the addition of silicone treatment, dry weight and protein content in the shoots and roots of both rice lines were increased, while the concentration of arsenic in these two organs was decreased. When seedlings were exposed to arsenic treatments, protein content, silicon concentration, and dry weight were decreased in both roots and shoots, while arsenic concentration was increased in both rice genotypes. The RNA-seq in DU-OE showed 5823 differentially expressed genes (DEGs), of which 2604 were up-regulated and 3219 down-regulated. Treatment of rice by arsenic and silicon has changed the expression of genes encoding cytokinin-responsive GATA transcription factor 1, protein IN2-1 homolog B, calcium-binding EGF domain-containing protein, Os01g0369700 protein, probable glutathione S-transferase GSTU1, glutathione S-transferase protein, Os09g0367700 protein, isocitrate dehydrogenase (NADP), and Os08g0522400 protein in the root of DU-OE. The present study’s findings showed that in the presence of silicon, the transgenic genotype is much more resistant to arsenic than the wild genotype of Dular rice.

## 1. Introduction

Heavy metals and metalloids are one of nature’s most persistent pollutants, and this property is one of the significant hazards facing the world. These metals do not decompose easily biologically and chemically; increasing the concentration of them in foods and the environment enhance their risk to the human body, including carcinogenesis, genetic mutations, bone damage, and metabolic and physiological effects such as kidney damage, lung problems, skin hazards, and others [1]. However, heavy metals in environmental pollution and their harmful effects on human health are not yet fully understood. Heavy metals enter the human body in various ways, and the most important pathway is eating foods, especially grains [2,3,4].

Arsenic (As) is a very toxic metalloid, which causes pollution in different countries around the world, like China, India, and the USA [5]. High levels of As in southeast Asian soils are due to the oxidation of sulfur-containing minerals in the Himalayas. These minerals form iron oxides when exposed to oxygen. Combining As with these iron oxides makes it more permeable to groundwater and soil [6]. As presented in several inorganic and organic kinds, the most important inorganic As kinds are dimethylarsinate (DMA) and methylarsonate (MMA), and the most important of organic kinds are arsenate (As(V)) and arsenite (As(III)) [6,7]. Groundwater is widely used in some areas for drinking, cooking, and irrigation of fields, and arsenic-contaminated water usage increases the concentration of arsenic in the soil and finally increases the uptake of arsenic in various plants [8].

When silicon (Si) combines with other elements, it constantly forms oxides or silicates and is absorbed as silicic acid in plants; it also participates in plant activities in the form of amorphous silica. Although Si is abundant in the earth’s crust, much of it is inaccessible to plants [9,10]. Si reduces heavy metals’ toxicity in plants by complicating and preventing the transfer of heavy metals from roots to shoots, breaking down heavy metal ions in plants, and stimulating antioxidant systems in plants [11].

According to the FAO, rice provides about 30% of energy and 20% of the protein needed by humans worldwide. Therefore, this crop is one of the most consumed grains in the food basket of 2.4 billion people worldwide, and the per capita consumption of this critical food production is about 58.8 kg per year [12,13].

Rice grows mainly in flooded conditions, and an anaerobic environment mainly increases the mobility and bioavailability of arsenic (As) [14]. There are two ways to uptake As in rice: (1) arsenite is one of the analogs of silicic acid and can be absorbed by plant roots via the silicic acid transport system; (2) arsenate is a chemical phosphate analog that plant roots can absorb through the phosphate transport protein systems [15]. Si consumption has been reported to decrease the concentration of arsenic in rice grains, stems, leaves, and husks and is also suitable for increasing rice plant growth, especially under biotic and abiotic stresses [16].

Research has shown that *Lsi1* and *Lsi2* are two carriers of Si in rice that also help transfer arsenic from the soil to rice [17]; therefore, As is transferred from the culture medium to rice through them [18]. Studies had shown that when Si was added to the culture medium, transgenic *Lsi1* or *Lsi1*-overexpression inhibitor rice types were more resistant to cadmium (Cd) toxicity. It also seems that increasing the resistance of rice to Cd stress depends not only on the amount of silicon in the culture medium, but also on the expression of *Lsi1* in rice [19]. The study has shown that suppressing the *Lsi1* gene reduces As concentrations in rice shoots and roots [20].

In the present study, we tried to identify the physiological and molecular mechanisms involved in the absorption of silicon and arsenic in Dular rice lines, and it was hypothesized that some genes are involved in regulating As stress resistance in rice plants. This experiment compared the expression changes of different genes by adding Si to the culture medium in As stress in two different lines of Dular rice (including Dular wild-type and Dular *Lsi1*-overexpressed rice).

## 2. Materials and Methods

### 2.1. Rice Cultivation

The best seeds were selected from Dular wild-type (DU-WT) and Dular *Lsi1*-overexpressed (DU-OE) rice genotypes, and then the grains were sterilized with 1% H_2_O_2_ for 15 min and immersed in deionized water for 48 h. Rice seeds were placed in a petri dish for four days in a culture room at 28 °C, and after germination, the best seedlings were selected and transferred for planting under hydroponic conditions. The pots (2.5 L) were filled with a solution prepared by Cock, Yoshida [21] method with some modification; by adding HCl or NaOH, the pH of the culture medium was adjusted to 5.8. The culture medium was renewed once a week, and when the seedlings had three leaves, different treatments were added to the culture medium.

The seedlings were exposed to four different treatments, which included the following: control (CK) (I), 30 μM As (II), 0.70 mM Si (III), and 30 μM As + 0.70 mM Si (IV). The amount of sodium added to the culture medium by Na_2_SiO_4_·9H_2_O was compensated by adding NaCl to the culture medium.

One and two weeks after adding different treatments to the culture medium, samples were collected. Then, the shoot samples were cleaned with distilled water, but the root samples were washed with 0.5 mM CaCl_2_ solution and then cleaned with distilled water. Finally, all samples were quickly transferred to a freezer at −10 °C until their physiological characteristics were specified. 

For transcriptomic analysis and quantitative RT-PCR, when seedlings had three leaves, they were treated with 30 μM As + 0.70 mM Si for three days. After collecting the seedlings, the root and shoot sections were separated and quickly transferred to a liquid nitrogen chamber at −196 °C. In this part of the experiment, DU-WT was considered as the control and DU-OE as the treatment.

The standard reference material used in this experiment was Guobiao standards (GB/T).

### 2.2. As Concentration Measurement

As concentration in different rice tissues were determined with Meharg and Jardineh methods [22]. Initially, 0.2 g of the samples were transferred to Kjeldahl tubes and combined with 1 mL of concentrated nitric acid. After the mixture was kept at room temperature for 24 h, 1 mL of H_2_O_2_ was added to the sample. Then for one hour, samples were exposed to a sand bath at 70 and 100 °C: The samples were cooled and filtered, and by adding distilled water, their volume was increased to 50 mL. Next, 1 mL of the filtered solution was mixed with 10% HCl, 5% potassium iodide, and 5% ascorbic acid, and with the addition of more distilled water, the volume increased to 10 mL. Finally, the concentration of As was measured with a hydride production apparatus using an atomic absorption spectrometry (Shimadzu 6200, Shimadzu Co., Kyoto, Japan) (FIG 100).

### 2.3. Dry Weight and Si Concentration Measurement

To dry the samples, they were exposed to 70 °C for two days. Then, for 20 min, the samples were pulverized and digested by 50% (*w*/*w*) NaOH at 121 °C [23]. The concentration of silicon in digested solution was specified through the colorimetric molybdenum blue method [24].

### 2.4. Soluble Protein Contents Measurement

The Bradford [25] method was used to measure the soluble protein content. Samples of plant material were powdered with liquid nitrogen using precooled mortars and pestles. Each sample supernatant and working standard solution were transferred to assay tubes. A blank containing 20 µL extraction buffer must also be prepared. Then, 1 mL of Bradford solution was combined with 20 μL of supernatant, and the adsorption of the mixture was measured at 595 nm after 5 min. The amount of soluble protein was determined by putting the number in the standard curve.

### 2.5. Transcriptomic Analyses

RNA was extracted from the roots of rice seedlings by the TRIZOL method [26], and the extracted RNA was then stored at −80 °C for use during the transcriptional sequencing. The extracted RNA was treated with RNase-free Dnase I; the mRNA was isolated from the total RNA using oligo (dT) to enrich it. Next, 1 µg of the separated mRNA was taken for the synthesis into double-stranded cDNA, which was synthesized using an oligo dT primer and SuperScript II, and following the manufacturer’s instructions was used for reverse transcription. According to the Ilumina pair sample preparation protocol, the double-stranded cDNA was shredded by nebulization and built the cDNA library. Analysis of differentially expressed genes (DEGs) was performed by using the DESeq R (1.10.1) package. Benjamini and Hochberg’s methods were used to obtain the *p*-value. Genes with significant errors were observed in values less than 0.05 [27]. For the gene ontology enrichment analysis (GO), a single enrichment analysis tool (SEA) was performed on the Viral Proteomic Tree Server (ViPTree), where y set the default parameters, and FDR set the threshold to *p* < 0.05.

### 2.6. Quantitative RT-PCR 

One microgram of the total RNA was subjected to reverse transcription using All-in-One First-Strand cDNA Synthesis SuperMix for qPCR (TransGen Biotech Co., Ltd. Beijing, China). Transcription levels of silicon-related genes and genes related to increasing rice resistance under arsenic stress were determined by quantitative RT-PCR. The sequence of primers used in this experiment is given in Table 1, and Actin-1(Os03g0718100) was used as the reference gene. Primers were designed for qRT-PCR analysis by online tools on the https://biodb.swu.edu.cn/qprimerdb/ (Accessed on 20 January 2020). The qRT-PCR reaction system was prepared by TransStart Tip Green qPCR SuperMix and an Eppendorf realplex4 instrument. The reaction process was as follows: initial denaturation at 94 °C for 30 s, denaturation at 94 °C for 5 s, annealing at 53 °C for 15 s, and extension at 72 °C for 10 s. When amplification was completed, product characteristics were determined based on the melting curve. Each candidate gene was performed with four independent reactions. The relative expression of the gene was calculated by the 2^−∆∆Ct^ method and by the threshold cycle values (Ct) of each candidate gene in both CK and experimental samples [28].

### 2.7. Statistical Analyses

The present experiment was performed in a completely randomized design with three replications. All findings were statistically analyzed by ANOVA test and SPSS 26.0 software following the LSD test (*p* ≤ 0.05).

It should be noted that two weeks after the As treatments to the culture medium, all DU-OE seedlings dried out.

## 3. Results

### 3.1. Arsenic Concentration of Shoots and Roots

**Figure 1:**
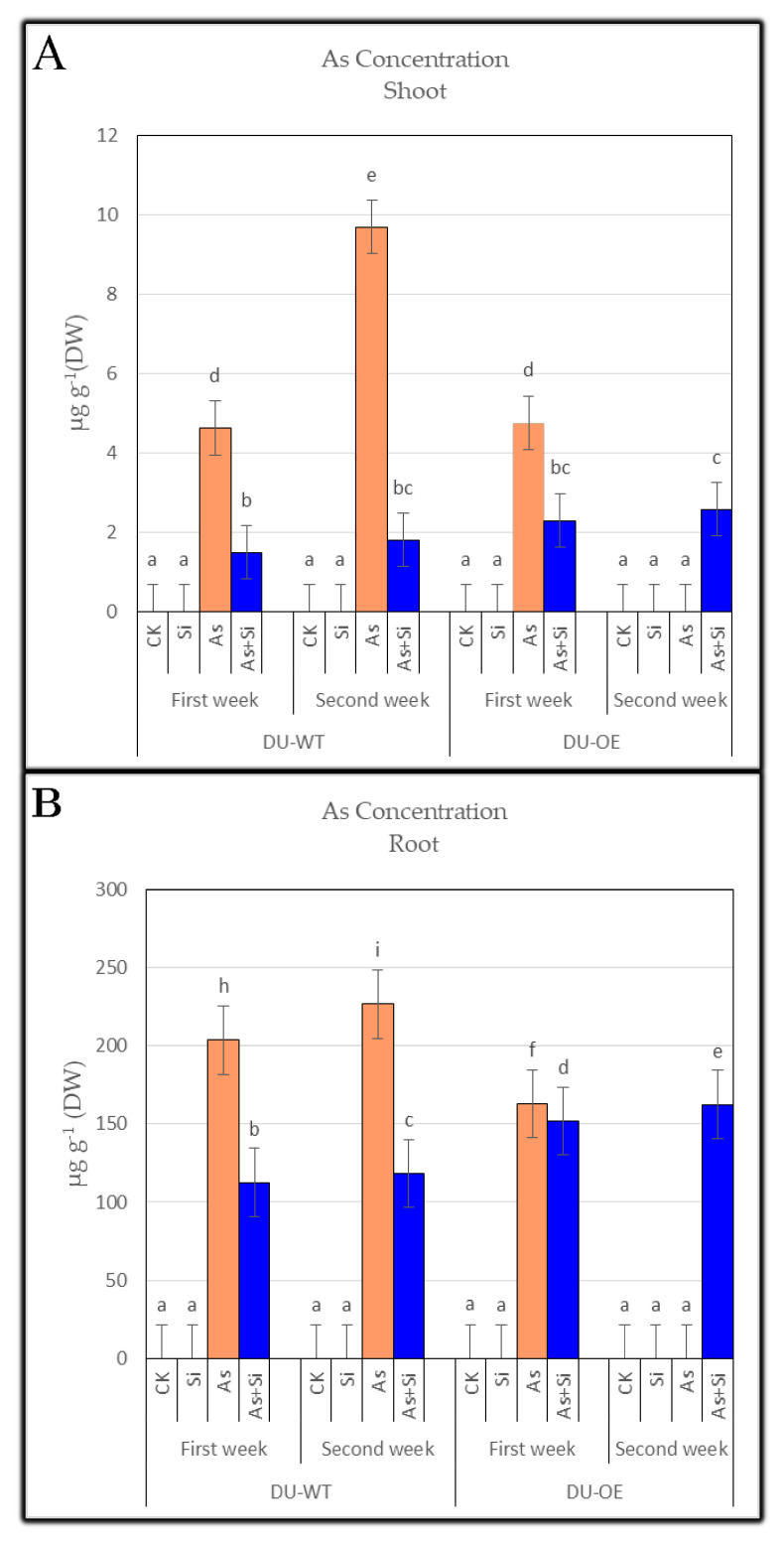
The arsenic concentration of DU-WT and DU-OE shoots, the first and second weeks after adding treatments (**A**). The arsenic concentration of DU-WT and DU-OE roots, the first and second weeks after adding treatments (**B**). The different letters on the bars indicate a significant difference among independent treatments (*p* ≤ 0.05); (DW: dry weight; CK: control; Si: 0.70 mM Si; As: 30 μM As; As+Si: 30 μM As + 0.70 mM Si).

When 30 μM As was added to the culture solution, arsenic accumulation in seedlings increased (Figure 1A,B). The results showed that shoot As concentrations in different rice genotypes increased compared to the control (*p* ≤ 0.05) when seedlings were exposed to As. The highest increase was seen in DU-WT shoots in the first and second weeks (Figure 1A), in which the highest concentrations of As were 4.62 µg g^−1^ DW and 9.68 µg g^−1^ DW in the first and second weeks, respectively. Additionally, the results of adding silicon (0.70 mM Si) to the medium were similar to the CK and equal to 0 µg g^−1^ DW of As. When 30 μM As + 0.70 mM Si were added to the culture medium, the concentration of As in shoots decreased comparing the addition of As treatment alone (30 μM As), and the highest significant decrease in the concentration of arsenic was seen in DU-WT shoots in both weeks. The lowest concentrations of arsenic were 1.49 µg g^−1^ DW and 1.8 µg g^−1^ DW in the first and second weeks, respectively.

The addition of arsenic treatment (30 μM As) increased the concentration of As in the roots of seedlings compared to the control (*p* ≤ 0.05). The highest increase was seen in the roots of DU-WT in both sampling periods, which were equal to 203.41 µg g^−1^ DW and 226.6 µg g^−1^ DW, respectively (Figure 1B). The present study showed that, when seedlings were treated only with 0.70 mM Si, the concentration of arsenic in the roots of both genotypes was 0 µg g^−1^ DW. However, adding 30 μM As + 0.70 mM Si to the culture solution significantly reduced the concentration of arsenic in the roots of both rice compared to the arsenic treatment alone (30 μM As), and the lowest As concentration belonged to the roots of DU-WT in both weeks at the values of 112.5 and 118.2 µg g^−1^ DW, respectively.

### 3.2. Silicon Concentration of Shoots and Roots

**Figure 2:**
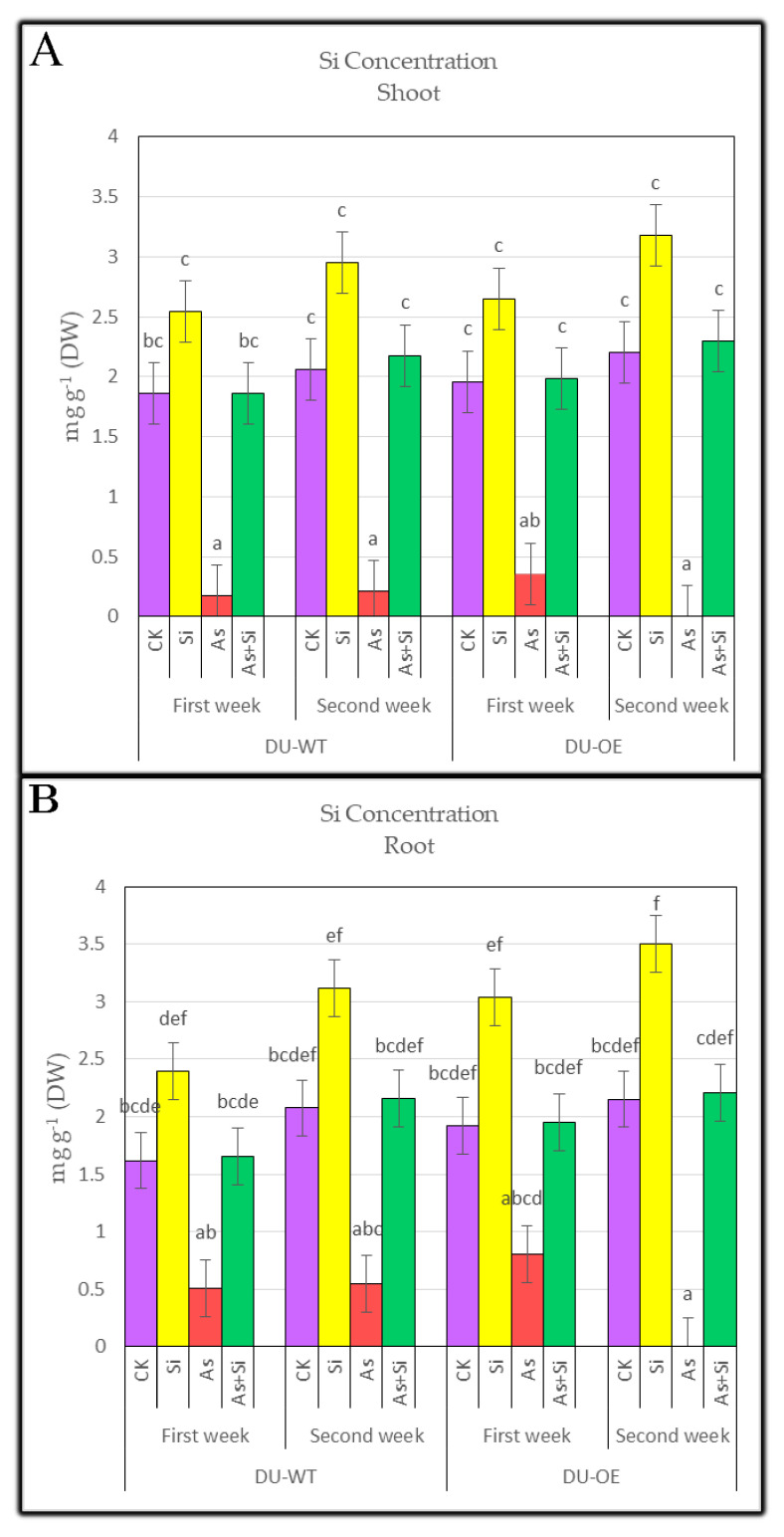
The Si concentration of DU-WT and DU-OE shoots, the first and second weeks after adding treatments (**A**); and the Si concentration of DU-WT and DU-OE roots, the first and second weeks after adding treatments (**B**). The different letters on the bars indicate a significant difference among independent treatment (*p* ≤ 0.05); (DW: dry weight; CK: control; Si: 0.70 mM Si; As: 30 μM As; As+Si: 30 μM As + 0.70 mM Si).

This study specified silicon concentration in the roots and shoots of both rice genotypes (DU-WT and DU-OE) under different treatments. (Figure 2A,B). The addition of 0.70 mM Si to the solution increased silicon concentration in the seedlings, but the Si concentration depended on the genotype. For example, the Si concentration in the shoots of DU-WT in both weeks after the addition of Si treatment was about 2.40 and 3.12 mg g^−1^ (DW), respectively, but in the DU-OE shoots, it was 2.65 and 3.18 mg g^−1^ (DW), respectively. Treatment of seedlings with arsenic significantly reduced the Si concentration in shoots of both genotypes; however, in the presence of As + Si treatment, the Si concentration of shoots increased compared to the application of arsenic treatment alone (30 μM As). When a30 μM As + 0.70 mM Si treatment was added to the culture medium, the highest increase in the Si concentration compared to the control (*p* ≤ 0.05) was seen in the shoots of DU-OE, and this increase compared to CK in both sampling periods was equal to 1.43% and 4.31%, respectively.

By adding 30 μM As to the culture solution, the silicon concentration in the rice roots decreased compared to the control (*p* ≤ 0.05). The most significant reduction in the Si concentration was seen in DU-WT roots in both weeks (Figure 2B), and it was equal to 68.60% and 73.80% in the first and second weeks, respectively. When seedlings were treated with 0.70 mM Si, the concentration of silicon in the roots of both genotypes increased significantly compared to the control, and Si concentration in DU-OE roots showed the highest increase compared to CK (*p* ≤ 0.05) in the first and second weeks (3.04 and 3.50 mg g^−1^ (DW), respectively). However, adding 30 μM As + 0.70 mM to the culture solution caused the highest increase in the Si concentration in DU-OE roots; that increase was equal to 1.51% and 2.41% compared to CK (*p* ≤ 0.05) in both sampling periods, respectively.

### 3.3. Dry Weight of Shoots and Roots

**Figure 3:**
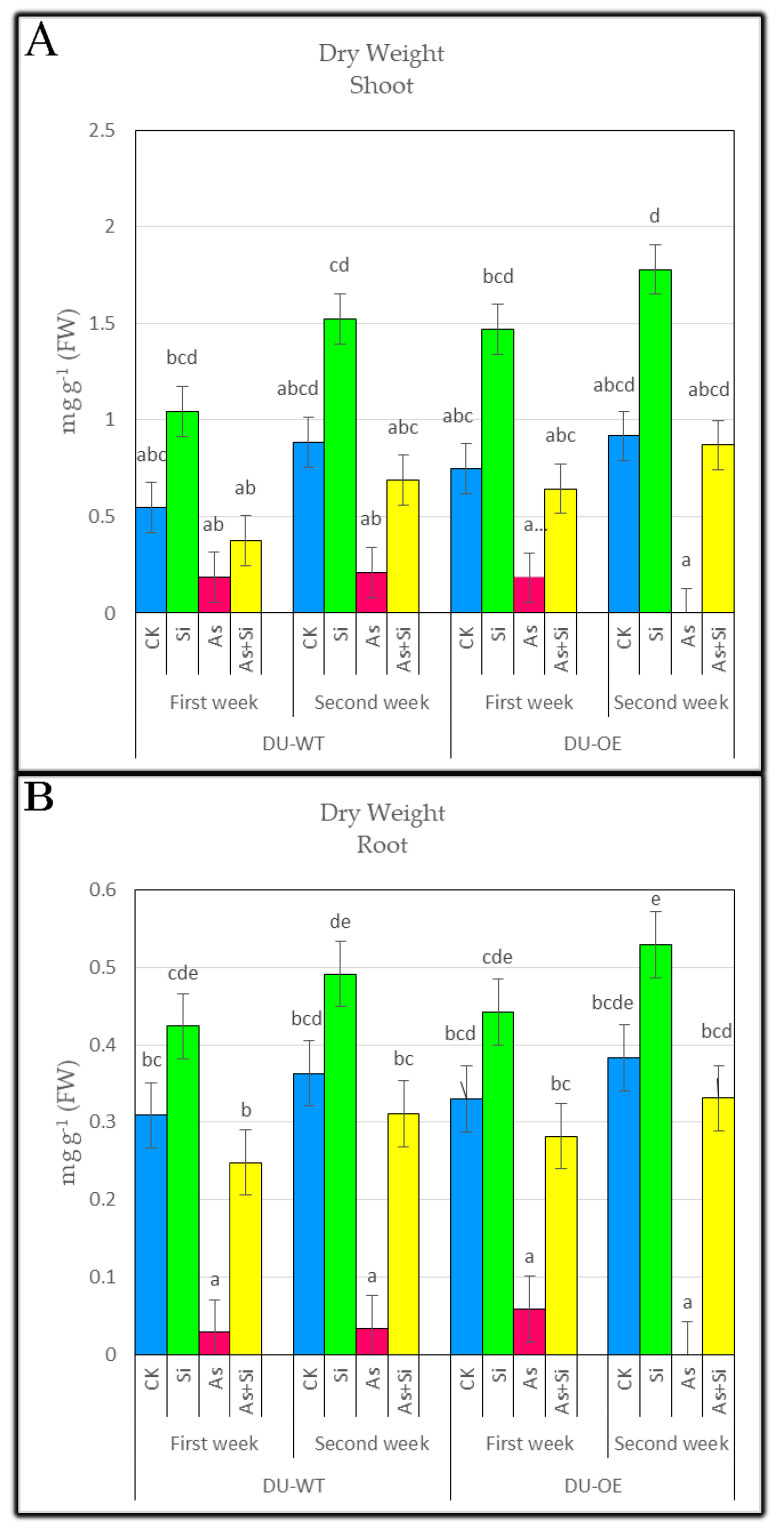
The dry weight of DU-WT and DU-OE shoots, the first and second weeks after adding treatments (**A**); the dry weight of DU-WT and DU-OE roots, the first and second weeks after adding treatments (**B**). The different letters on the bars indicate a significant difference among independent treatment (*p* ≤ 0.05); (FW: fresh weight; CK: control; Si: 0.70 mM Si; As: 30 μM As; As+Si: 30 μM As + 0.70 mM Si).

Further studies on 30 μM As treatment showed that adding this treatment to the medium significantly reduced the dry weight in the shoots and roots of both rice genotypes in both weeks, and DU-OE roots and shoots showed the higher dry weight loss compared to DU-WT (Figure 3A,B; Table 2). However, the dry weight of DU-OE roots and stems showed a higher increase in the presence of Si treatment. By adding 0.70 mM Si to the culture medium, the dry weight of DU-OE shoots and roots in both sampling periods increased by 97.05% and 94.11% and 33.93% and 38.12% compared to CK, respectively, but this increase in DU-WT shoots and roots was equal to 90.69% and 72.12% and 37.21% and 35.26%, respectively. (Figure 3A,B; Table 2). 

The dry weight of seedlings treated with 30 μM As + 0.70 mM Si in both rice genotypes decreased compared to CK, especially in the case of the DU-OE genotype, where it was shown that the addition of Si increases the biological tolerance threshold and controls the uptake and transfer of As in roots and shoots, which indicates the antagonism between arsenic and silicon by the existing *Lsi1* in rice (Figure 3A,B; Table 2). 

### 3.4. Soluble Protein Contents of Shoots and Roots

**Figure 4:**
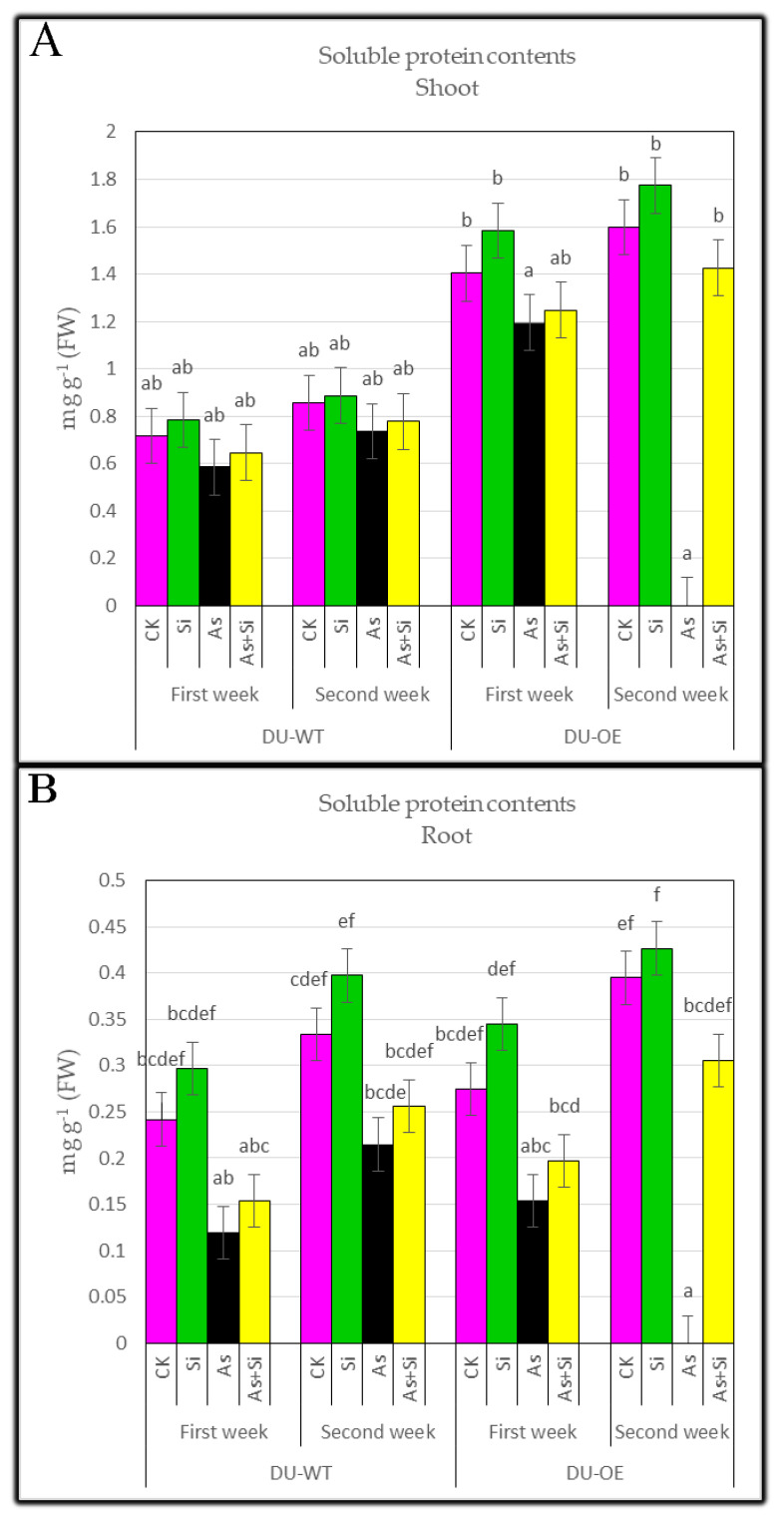
Soluble protein contents of DU-WT and DU-OE shoots, the first and second weeks after adding treatments (**A**); soluble protein contents of DU-WT and DU-OE roots, the first and second weeks after adding treatments (**B**). The different letters on the bars indicate a significant difference among independent treatment (*p* ≤ 0.05); (FW: fresh weight; CK: control; Si: 0.70 mM Si; As: 30 μM As; As+Si: 30 μM As + 0.70 mM Si).

Data analysis showed that adding 0.70 mM Si to the culture solution increased the soluble protein content in rice lines’ shoots in both sampling periods (Figure 4A). The highest increase in soluble protein content was seen in the shoots of DU-OE compared to CK (*p* ≤ 0.05), which was equal to 1.58 and 1.77 mg g^−1^ FW in both weeks, respectively. The arsenic-containing treatment (30 μM As) reduced the soluble protein content of shoots compared to the control (*p* ≤ 0.05), and the highest significant decrease was seen in DU-WT shoots in both weeks, which was equal to 0.58 and 0.73 mg g^−1^ FW, respectively. When adding 30 μM As + 0.70 mM Si treatments, the soluble protein content in both rice genotypes reduced compared to the control (*p* ≤ 0.05), and the highest reduction was related to DU-WT shoots, which showed a decrease of 9.78% and 9.25% in the first and second weeks compared to the CK, respectively.

Treating seedlings with silicon (0.70 mM Si) significantly increased soluble protein content in roots compared to CK. The highest increase in soluble protein content in DU-OE roots was observed, equal to a 25.58% and 8.08% increase in both weeks, respectively (Figure 4B). By adding arsenic treatment (30 μM As) to the culture medium, the soluble protein content of both rice roots was reduced compared to CK (*p* ≤ 0.05), and the highest significant decrease compared to CK was seen in DU-WT roots in the first and second weeks, which were equal to 0.12 and 0.21 mg g^−1^ FW, respectively. When seedlings were exposed to 30 μM As + 0.70 mM Si, the greatest reduction in soluble protein content was observed in DU-WT roots compared to CK (*p* ≤ 0.05), and it showed a decrease of 36.28% and 23.27% in both sampling periods, respectively.

### 3.5. Transcriptome Analysis of Rice Roots

**Figure 5:**
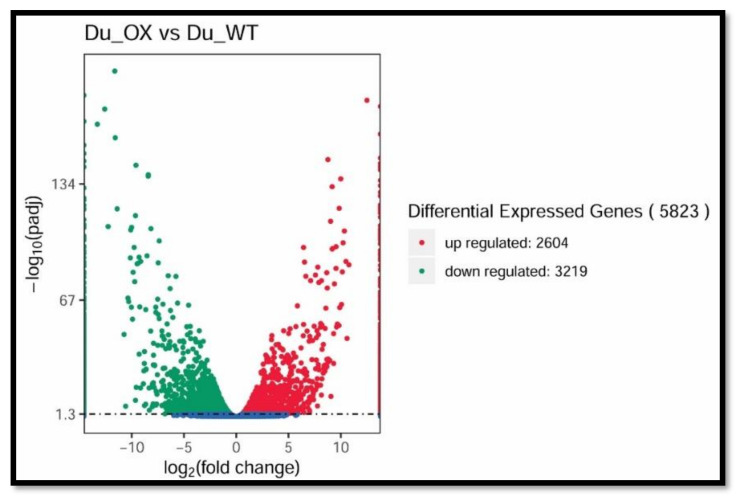
Differentially expressed genes of DU-OE as compared with DU-WT (including up-regulated and down-regulated genes).

Illumina high-throughput sequences related to arsenic and silicon in rice were used, and for this purpose, seedlings were treated with 30 μM As + 0.70 mM Si, and the coefficient was calculated to evaluate their reproducibility. Using DESeq, differentially expressed genes (DEGs) were known between DU-WT (CK) and DU-OE (Figure 5).

**Figure 6:**
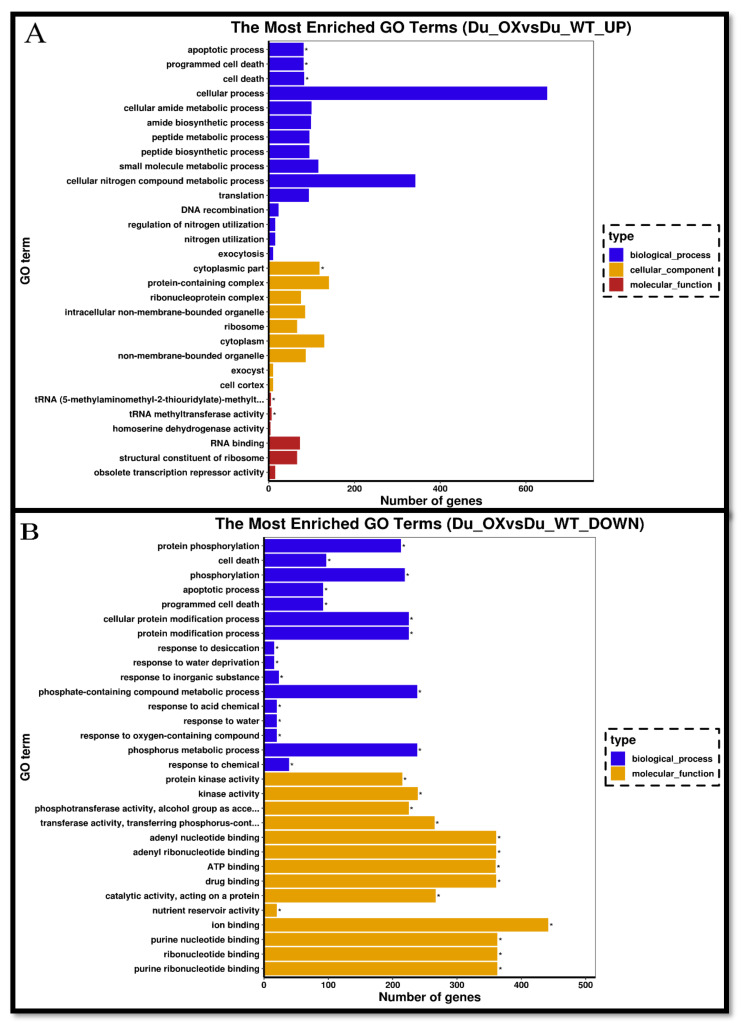
GO classification of up-regulated genes in the DU-OE line (**A**) and GO classification of down-regulated genes in the DU-OE line (**B**).

The list of DEGs for DU-OE included 5823 genes, including 2604 up-regulated genes and 3219 down-regulated genes. We used ViPTree to classify GO on the expressed genes affected by As and Si, and enriched GO terms categorized the cellular component, molecular function, and biological process. GO enrichment conditions of the biological process (GO: 0008152) were mainly related to the cellular process (GO: 0009987) and metabolic process (GO: 0008152) and response to stimulus (GO: 0050896). In the classification of the molecular function (GO: 0003674), the enriched DEGs were related to binding (GO: 0005488), catalytic activity (GO: 0003824), and nutrient reservoir activity (GO: 0045735), while enriched DEGs in the cellular component (GO: 0005575) were related to the cell (GO: 0005623) and cell parts (GO: 0044464) (Figure 6A,B).

Kyoto Encyclopedia of Genes and Genomes (KEGG) enrichment analysis in DU-OE found that DEGs in RNA degradation contained 15 genes, of which 12 genes are up-regulated and 3 are down-regulated; while DEGs in ribosome included 46 genes, of which 7 are up-regulated and 3 are down-regulated. KEGG enrichment analysis also showed that DEGs involved in purine metabolism included 22 genes, of which 15 are up-regulated genes and 7 are down-regulated, while DEGs in glutathione metabolism contained 17 genes, of which 11 genes are up-regulated and 6 are down-regulated.

KEGG enrichment analysis showed that DEGs in cysteine and methionine metabolism contained 21 genes, of which 14 are up-regulated and 6 are down-regulated; however, DEGs in carbon metabolism included 33 genes, of which 22 genes are up-regulated and 11 are down-regulated. KEGG enrichment analysis also showed that DEGs in the biosynthesis of secondary metabolites contained 135 genes, of which 74 are up-regulated and 61 are down-regulated genes, but DEGs in the biosynthesis of amino acids contained 36 genes, of which 22 genes are up-regulated and 14 are down-regulated; on the other hand, DEGs in starch and sucrose metabolism contained 19 genes, of which 2 genes are up-regulated and 17 are down-regulated. 

KEGG enrichment analysis showed that DEGs in plant hormone signal transduction included 25 genes, of which 7 are up-regulated and 18 are down-regulated, but DEGs in phenylpropanoid biosynthesis contained 21 genes, of which 7 genes are up-regulated and 18 are down-regulated, while DEGs in metabolic pathways include 229 genes, of which 7 are up-regulated and 18 are down-regulated. 

**Figure 7:**
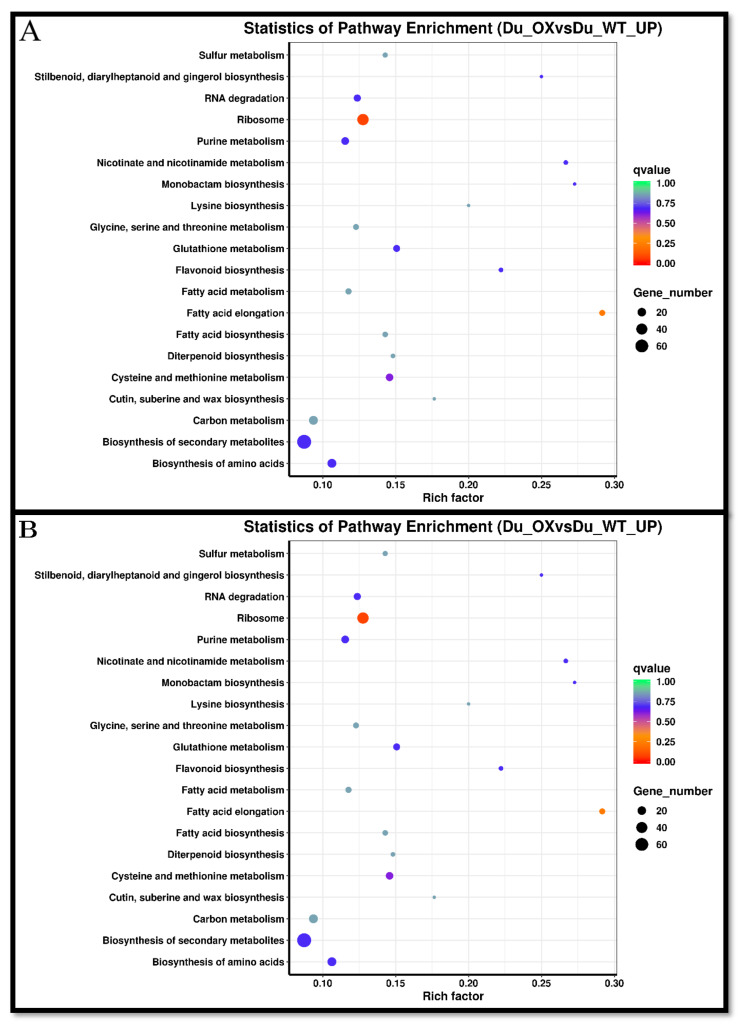
Kyoto Encyclopedia of Genes and Genomes pathway enrichment scatter plot of up-regulated genes in the DU-OE line (**A**) and Kyoto Encyclopedia of Genes and Genomes pathway enrichment scatter plot of down-regulated genes in the DU-OE line (**B**).

Finally, it was found that the factors that cause rice resistance to arsenic are only regulated by the expression patterns of these genes (Figure 7A,B).

### 3.6. Expression of Some Genes in DU-OE Exposed to Si and As

**Figure 8:**
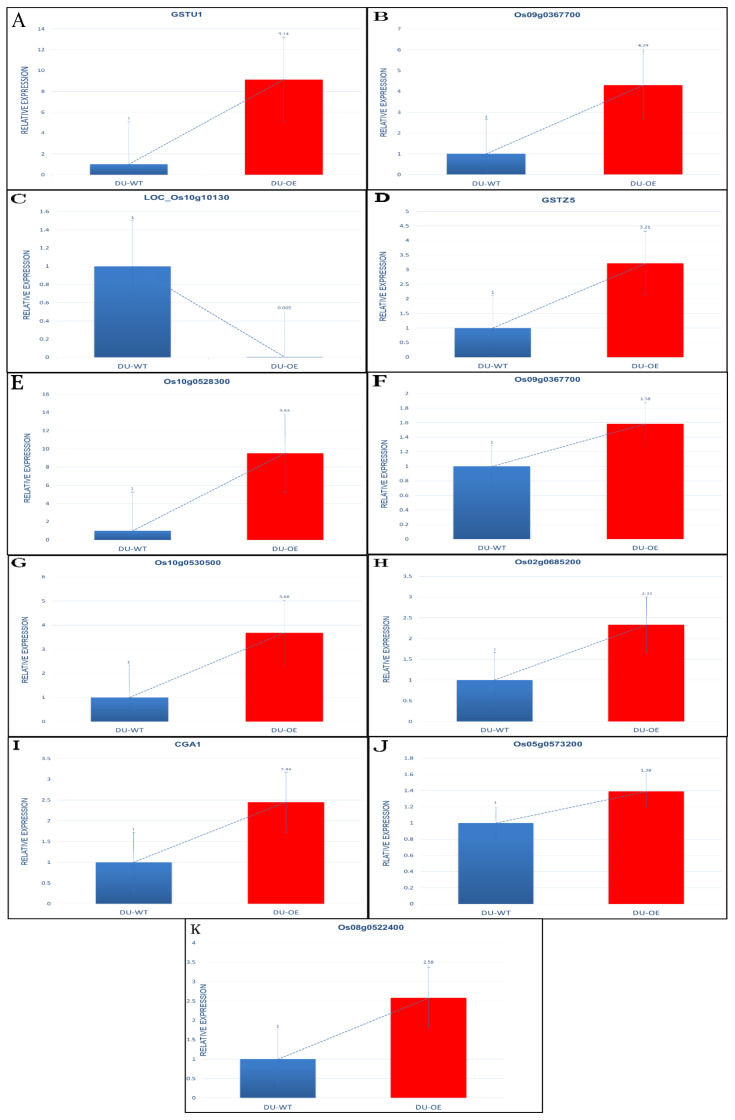
Fold change of gene expression levels in DU-OE as compared to DU-WT. GSTU1 (**A**); Os09g0367700 (**B**); LOC_Os10g10130 (**C**); GSTZ5 (**D**); Os10g0528300 (**E**); Os09g0367700 (**F**); Os10g0530500 (**G**); Os02g0685200 (**H**); Cga1 (**I**); Os05g0573200 (**J**); Os08g0522400 (**K**).

The expression of probable glutathione S-transferase GSTU1, which regulates glutathione transferase activity in rice roots, showed that it was up-regulated when seedlings were treated with arsenic and silicon (Figure 8A). 

The Os01g0367700 protein, which regulates glutathione transferase activity in rice, was up-regulated when the seedlings were treated with arsenic and silicon (0.43 fold), but the expression of the IN2-1 protein, as putatively expressed (regulates protein glutathionylation), was down-regulated (6.63 fold; Figure 8B,C). 

The role of protein IN2-1 homolog B in rice plants is regulated by protein glutathionylation. QRT-PCR showed that the expression of GSTZ5 in DU-OE was up-regulated compared to CK, and the results also showed that the expression of glutathione S-transferase protein (Os10g0528300), which regulates glutathione transferase activity and a glutathione metabolic process in rice, was highly up-regulated (Figure 8D,E).

Os09g0367700 protein, which regulates glutathione transferase activity in DU-OE rice, was up-regulated when the roots were treated by arsenic and silicon (Figure 8F).

The expression of Os10g0530500 protein, which regulates glutathione transferase activity in DU-OE rice, was up-regulated compared to the control, and the expression of Os02g0685200 protein in the presence of arsenic and silicon treatment was also highly up-regulated compared to CK (Figure 8G,H).

The effect of As and Si treatments on the gene expression of cytokinin GATA transcription factor1 (*Cga1*) in DU-OE was highly up-regulated compared to control (Figure 8I).

Isocitrate dehydrogenase (NADP) (Os05g0573200), which regulates catalytic activity in DU-OE, was highly up-regulated (Figure 8J).

The expression of Os08g0522400 protein, which regulates peroxidase activity and oxidative stress in DU-OE rice, was highly up-regulated (Figure 8K).

## 4. Discussion

Si decreases the toxicity of some metalloids and heavy metals in plants by building complexes, preventing heavy metals from the roots to the aerial parts of plants, dividing metal ions, and activating plant antioxidant systems [11]. Si can also reduce the uptake and transport of metals in rice. Various researchers have reported that using silicon increases plant tolerance to the toxicity of metals by decreasing the uptake of heavy metals into plant roots, reducing their transfer to stems, and ultimately reducing their toxicity to plant tissues [29,30]. The present study also showed that the addition of Si increases the silicon concentration in rice roots and shoots, while decreasing the As concentration in Dular rice roots and shoots [11]. Seyfferth and Fendorf [16] also reported that, by adding Si to the soil pore water, the concentration of arsenic in rice grains was significantly reduced. Numerous similar studies have shown that As and Si compete for uptake by the root of plants, and increasing one reduces the other [16,30]. In addition, Si significantly affects the concentration of arsenic in various rice organs such as stems, leaves, grains, and husks [31]. Furthermore, different studies have shown that applying Si in different methods, such as spraying and adding to the soil, reduces the concentration of different inorganic and organic species of arsenic in different organs of rice plants [32,33]. Rice is a silicon collector, and concentration of silicon can reach 100 g/kg in rice shoots; however, because Si has similar transport pathways to arsenic (*Lsi1* and *Lsi2*), the addition of As or Si to the culture medium can affect the uptake of the other in rice and eventually reduce its accumulation in different tissues of the rice plant [18,34]. Different studies have shown that different rice lines have different potentials for the uptake Si and As, and their tolerance to As toxicity is also different [35]. Some researchers have reported that adding similar levels of As to the culture medium of different rice genotypes causes different levels of arsenic to accumulate in those rice lines [35], similar to the present study’s results.

This study also showed that the addition of Si to the culture medium increases the amount of soluble protein in different rice, and it has been seen that silicon has a unique role against ROS toxicity, decreasing oxidative damage, decreasing protein degradation, reducing stress, improving protein synthesis, and improving protein stress-induced metabolism [36,37]. Some studies have shown that the addition of As to the culture medium reduces the soluble protein content. It may be due to the reaction of arsenic with sulfhydryl groups of proteins (severely inhibits photosynthetic activity and rice growth), increasing the rate of destruction by disrupting the membrane system, causing further oxidative damage (inhibits protein synthesis), and reducing the level of As (V) to As (Ⅲ) in cells responsible for protein damage through oxidation of thiol groups [36,38].

This study showed that the genes related to metabolic pathways were expressed after the addition of arsenic to the culture medium, and as we know, metabolic pathways are regulated by various physiological processes necessary for plant growth and development [39]. Our research also found that after exposure to stress, transgenic rice reacted differently to gene expression and antioxidant defense, and some studies have provided new insights into plant defense mechanisms, gene regulation, and gene networks in response to toxicity. One of the most important findings is the effect of As on the differential expression of the gene encoding glutathione-S-transferases (GSTs), one of the essential mechanisms in plants against As toxicity [40], which is the same as the results of our study. Different studies on various plants, including rice and Indian mustard, have shown that the overexpression of some genes makes transgenic species more resistant to As and Cd [19].

The present study deals with the differences in susceptibility among two rice lines to arsenic. This study tried to specify the effect of GST and other antioxidative enzyme genes to reduce As pollution and the positive effect of Si on reducing contamination. In this experiment, qRT-PCR showed that the number of genes related to GST was significantly expressed. Those genes included GSTU1, Os01g0369700, LOC_Os03g17460, GSTZ5, Os10g0528300, Os09g0367700, and Os10g0530500. GSTs were found in most eukaryotes, prokaryotes, and aerobic protect cells against toxic chemicals and stress [41]. The decrease and increase of antioxidative enzymes due to different stresses cause the reduction and increase of H_2_O_2_ in the plants and show that these genes’ expression is different in diverse varieties of plants [42].

*Lsi1* is an active transmitter found mainly in the central region of rice root cells, which transmits Si and As (III) into the rice roots; therefore, the transmission route of As (III) from the culture medium to rice is mediated by *Lsi1* [18]. Another study showed that Si is transmitted from the root epidermis to the root stele through *Lsi1* and *Lsi2* [43]. The expression of the *Lsi1* gene in rice increases the resistance of plants to arsenite treatments [44], and studies have shown that transgenic *Lsi1* or *Lsi1*-overexpressor transgenic rice is more resistant to toxicity of Cd by adding Si. It seems that increasing resistance to Cd stress depends not only on the amount of silicon in the culture medium, but also on the *Lsi1* expression of rice [19]. Research has shown that suppressing the *Lsi1* gene effects reduces the accumulation of arsenic in rice roots and shoots [20].

## 5. Conclusions

This experiment showed a direct relationship between the accumulation of arsenic and silicon in rice shoots and roots and the addition of these elements to the culture medium. This study also showed that the addition of silicon to the culture medium reduces the accumulation of arsenic in various tissues of the DU-OE line more than DU-WT. However, if silicon is not added to the culture medium, the DU-OE line will be severely affected by arsenic toxicity, especially in the second week, causing the plant to dry completely. The results of RNA-Seq analysis showed that different genes are expressed that belong to different gene families, suggesting that rice has complex pathways to arsenic stress conditions. Therefore, it is recommended to plant transgenic rice lines in arsenic-contaminated environments and add silicone fertilizers to the culture medium to reduce the amount of arsenic-contaminated rice as much as possible, especially in the early planting periods, which ultimately increases the health of human society. It is also recommended that further studies be performed to identify the physiological and molecular mechanisms involved in the uptake, transport, and accumulation of arsenic and silicon in different rice lines.

## Figures and Tables

**Table 1:** All gene primers were used for qRT-PCR.

Gene Name	Protein Name	Primer Sequences	Amplicon (bp)
GSTU1-F	Probable glutathione S-transferase GSTU1	GTGAGTTTGTTGTTACCGTTGA	84
GSTU1-R		GGACTCGTCAGTGTTGTACATA	
Os01g0369700-F	Os09g0367700 protein	GTTCGGTGAGATTCCAGTACTG	86
Os01g0369700-R		TGTACTTGCGAAAGATGTACCT	
LOC_Os03g17460-F	IN2-1 protein, putative, expressed	CACCTTGATCTGCATTTGTCAA	89
LOC_Os03g17460-R		ATGGGCAAATGTTGACATGTAC	
GSTZ5-F	Protein IN2-1 homolog B	AAGATTGTCGCGATTGATCTTG	95
GSTZ5-R		TGATTGTTGTGCTCAAGTGAAG	
Os10g0528300-F	Glutathione S-transferase	CAAGATCTTCGACGAGGAGAAG	123
Os10g0528300-R		CTCATCTTAGCGAACTCGACC	
Os09g0367700-F	Os09g0367700 protein	TGGTTCCACGCCTACGAGA	126
Os09g0367700-R		CTTCTCAGGATCAGTAAGCGTC	
Os10g0530500-F	Os10g0530500 protein	ACAACATGTTCCCTGGAATGG	166
Os10g0530500-R		TCGACGTACCCGATGGAGTC	
Os02g0685200-F	Os02g0685200 protein	CGGTGGGTTCTCGAATAACTC	193
Os02g0685200-R		CGTGGTTGCAATTGACATCTTA	
CGA1-F	CYTOKININ-RESPONSIVE GATA TRANSCRIPTION FACTOR 1	CACAGAACCCGATATCCAAGG	197
CGA1-R		GCCCTCATCAAATTAACGGTAC	
Os05g0573200-F	Isocitrate dehydrogenase [NADP]	CTAAATGGCACTGTGTTCAGAG	252
Os05g0573200-R		GGACTCGTCAGTGTTGTACATA	
Os08g0522400-F	Os08g0522400 protein	CAAAGACAAGCTTTCACCGTAA	282
Os08g0522400-R		CAGAAAAGAACGCTGCCTTTAA	
actin1(Os03g0718100)-F		CTTCATAGGAATGGAAGCTGCGGGTA	26
actin1(Os03g0718100)-R		CGACCACCTTGATCTTCATGCTGCTA	

**Table 2:** ANOVA results due to As, Si, rice genotypes, and their interactions on the As concentration and dry weight of DU-WT and DU-OE shoots and roots at different sampling times.

Sampling Time	Parameter	Genotypes	As	Si	Genotypes × As	Genotypes × Si	As × Si	Genotypes × As × Si
1st week	Shoot As concentration	42.01 **	16595.59 **	2453.05 **	118.54 **	50.85 **	904.34 **	15.45 **
	Root As concentration	22.18 **	10391.57 **	711.33 **	5.58 **	46.15 **	207.94 **	92.80 **
	Shoot dry weight	17.21 **	55.04 **	22.71 **	0.70	1.42	2.11	0.26
	Root dry weight	3.21	43.13 **	23.79 **	0.02	0.38	1.24	0.13
2nd week	Shoot As concentration	863.84 **	12289.71 **	2397.37 **	611.74 **	799.68 **	1161.99 **	371.13 **
	Root As concentration	32.77 **	12514.50 **	866.25 **	12.56 **	148.88 **	217.80 **	123.61 **
	Shoot dry weight	2.87 **	45.87 **	28.03 **	0.06	0.25	1.20	0.30
	Root dry weight	0.86	44.26 **	26.90 **	0.02	0.06	1.66	0.03

** Significant at the level of 1%.

## Data Availability

All data generated during this study are included in this published article, and the raw data used or analyzed during the current study are available from the corresponding author on reasonable request.
